# Presynaptic CRF1 Receptors Mediate the Ethanol Enhancement of GABAergic Transmission in the Mouse Central Amygdala

**DOI:** 10.1100/tsw.2009.1

**Published:** 2009-01-18

**Authors:** Zhiguo Nie, Eric P. Zorrilla, Samuel G. Madamba, Kenner C. Rice, Marissa Roberto, George Robert Siggins

**Affiliations:** ^1^ Burnham Institute for Medical Research, La Jolla, CA, United States; ^2^ Committee on the Neurobiology of Addictive Disorders, Alcohol Research Center, Scripps Research Institute, San Diego, CA, United States; ^3^ Department of Molecular and Integrative Neurosciences, Alcohol Research Center, Scripps Research Institute, San Diego, CA, United States; ^4^ Chemical Biology Research Branch, National Institute on Drug Abuse National Institute on Alcohol Abuse and Alcoholism, National Institutes of Health, Bethesda, MD, United States

**Keywords:** electrophysiology, alcohol, gamma aminobutyric acid, corticotrophin-releasing factor, corticotropin-releasing hormone, CRH, urocortin, stresscopin, whole-cell patch, IPSC

## Abstract

Corticotropin-releasing factor (CRF) is a 41-amino-acid neuropeptide involved in stress responses initiated from several brain areas, including the amygdala formation. Research shows a strong relationship between stress, brain CRF, and excessive alcohol consumption. Behavioral studies suggest that the central amygdala (CeA) is significantly involved in alcohol reward and dependence. We recently reported that the ethanol augmentation of GABAergic synaptic transmission in rat CeA involves CRF1 receptors, because both CRF and ethanol significantly enhanced the amplitude of evoked GABAergic inhibitory postsynaptic currents (IPSCs) in CeA neurons from wild-type (WT) and CRF2 knockout (KO) mice, but not in neurons of CRF_1_ KO mice. The present study extends these findings using selective CRF receptor ligands, gene KO models, and miniature IPSC (mIPSC) analysis to assess further a presynaptic role for the CRF receptors in mediating ethanol effects in the CeA. In whole-cell patch recordings of pharmacologically isolated GABAAergic IPSCs from slices of mouse CeA, both CRF and ethanol augmented evoked IPSCs in a concentration-dependent manner, with low EC_50_s. A CRF_1_ (but not CRF_2_) KO construct and the CRF_1_-selective nonpeptide antagonist NIH-3 (LWH-63) blocked the augmenting effect of both CRF and ethanol on evoked IPSCs. Furthermore, the new selective CRF_1_ agonist stressin_1_, but not the CRF_2_ agonist urocortin 3, also increased evoked IPSC amplitudes. Both CRF and ethanol decreased paired-pulse facilitation (PPF) of evoked IPSCs and significantly enhanced the frequency, but not the amplitude, of spontaneous miniature GABAergic mIPSCs in CeA neurons of WT mice, suggesting a presynaptic site of action. The PPF effect of ethanol was abolished in CeA neurons of CRF_1_ KO mice. The CRF_1_ antagonist NIH-3 blocked the CRF- and ethanol-induced enhancement of mIPSC frequency in CeA neurons. These data indicate that presynaptic CRF_1_ receptors play a critical role in permitting or mediating ethanol enhancement of GABAergic synaptic transmission in CeA, via increased vesicular GABA release, and thus may be a rational target for the treatment of alcohol abuse and alcoholism.

## INTRODUCTION

Despite decades of research, the cellular and molecular mechanisms underlying the intoxicating and addictive properties of ethanol are not well understood. Synapses are considered to be the most sensitive site for central ethanol effects[2], in part because ethanol interacts with a number of transmitter-gated ion channels at relatively low concentrations. Postsynaptic γ-aminobutyric acid-A ($GABA_A$) receptors have received by far the most attention in this regard[2,3,4,5,6,7]. Generally, acute ethanol enhances $GABA_A$ergic function in several central nervous system (CNS) areas, such as the nucleus accumbens, hippocampus, ventral tegmental area (VTA), and central amygdala (CeA). However, much recent evidence has shown that ethanol can also enhance GABAergic transmission presynaptically by enhancing GABA release[2,3,5,7,8,9].

Corticotropin-releasing factor (CRF) and its paralogs urocortins 1, 2, and 3 are involved in stress responses, and are implicated in stress-related disorders, such as anxiety and depression[10,11,12,13,14]. CRF functions as a hormone in the hypothalamic-pituitary axis, releasing adrenocorticotropic hormone (ACTH) from the anterior pituitary, and as a neurotransmitter in the CNS, mediating numerous behavioral stress responses. The CeA contains CRF receptors and abundant CRF-containing fibers[15,16](E. Crawford and G.R. Siggins, unpublished data); CRF itself is generally colocalized in CeA neurons together with GABA[17,18]. Increased release of CRF can be measured in the CeA under conditions of acute stress[19,20,21,22] and decreases in stress reactions result when CRF receptor antagonists are injected into the CeA[20]. Direct electrical stimulation of, or microinjection of CRF into, the CeA produces physiological effects much like the stress response[23]. CRF-deficient mice show increased voluntary ethanol consumption following stress[24].

Low CRF concentrations can influence neuronal properties in the CNS (see, e.g., [25,26]). CRF decreases the slow afterhyperpolarizing potential in the hippocampus[25] and CeA[27], and enhances R-type voltage-gated calcium channels in rat CeA neurons[28]. These and other data[1,29,30] also suggest that CRF plays an important role in regulating synaptic transmission in the CNS. For example, in VTA dopamine neurons, CRF potentiates N-methyl-D-aspartic acid (NMDA)–mediated synaptic transmission via $CRF_2$ activation[30], and we recently found that CRF augments GABAergic inhibitory transmission in mouse CeA neurons via $CRF_1$ activation[1].

The CeA is a brain region intimately involved in alcohol dependence and excessive drinking[31,32]. During ethanol withdrawal, rats manifest behavioral analogues of anxiety correlated with increased CRF-like immunoreactivity in dialysates of the amygdala[19] and corresponding reductions in amygdala tissue content[33]. These behaviors are blocked by CRF antagonists injected into the CeA[34,35]. GABA is the main inhibitory neurotransmitter in the adult mammalian CNS, including the rodent CeA. Behavioral studies have shown that a $GABA_A$ antagonist injected into the amygdala can decrease ethanol self-administration in dependent rats[36], and that CRF systems in the amygdala are activated during ethanol withdrawal[19,37].

In our laboratory, electrophysiological data showed that ethanol enhanced GABAergic transmission at both pre- and postsynaptic sites in a rat CeA slice[5], and that a similar ethanol augmentation of GABA inhibitory postsynaptic currents (IPSCs) in mouse CeA required activation of $CRF_1$ receptors[1]. These combined data suggest that interactions between the CRF and GABAergic systems in the CeA may play an important role in alcohol reward and dependence. Therefore, in the present study we have further explored the cellular site of CRF action and the receptor subtype involved in this interaction of ethanol with the CeA GABAergic system. Here, we report that ethanol and CRF both enhance GABAergic transmission via presynaptic $CRF_1$ receptor activation in the mouse CeA.

## MATERIALS AND METHODS

### Slice Preparation

We prepared amygdala slices from male C57Bl/6J mice as partly described previously[1], with slight modifications. All experiments were performed in accord with The Scripps Research Institute IACUC and NIH guidelines on the care and use of laboratory animals. Briefly, male mice (5–10 weeks old) were anesthetized with halothane (4%) and decapitated, and the brains rapidly removed into ice-cold artificial cerebrospinal fluid (ACSF) gassed with 95% $O_2$/5% $CO_2$ and containing (in m*M*): 130 NaCl, 3.5 KCl, 1.25 $NaH_2PO_4$, 1.5 $MgSO_4.7H_2O$, 2.0 $CaCl_2$, 24 $NaHCO_3$, and 10 *d*-glucose. We cut transverse slices 400 μm thick on a Leica VT 1000S vibrating cutter (McBain Instruments, Chatsworth, CA) and transferred them to a beaker containing ACSF bubbled with 95% $O_2$/5% $CO_2$ at room temperature. After incubation for at least 90 min, slices were transferred to the recording chamber. During recordings, slices were submerged and continuously superfused with ACSF at a rate of 2 ml/min. We performed most recordings at room temperature (~24°C), although we performed some experiments at 32°C. There were no differences for ethanol and CRF effects between slices held at 24 vs. 32°C.

### Whole-Cell Recording

We made whole-cell patch-clamp recordings with pipettes pulled on a Sutter Instruments puller from borosilicate glass and containing (in m*M*): CsCl 130, HEPES 10, EGTA 10, $MgCl_2$ 1.0, and ATP 2.0, adjusted to pH 7.2–7.4 with CsOH 1.0. Recording electrode resistance was 3–5 MΩ. In most experiments, we visualized CeA neurons using an upright Olympus microscope with infrared illumination and Nomarski optics. We recorded neurons (mostly in the medial part of the CeA) using an Axopatch 200B amplifier (Molecular Devices, Sunnyvale, CA) and stored the data for later analysis using pClamp software (Molecular Devices). We clamped cells at –70 mV and monitored the series resistance continuously using small (10 mV) hyperpolarizing voltage steps (200 msec). We studied only those cells demonstrating <20 MΩ series resistance.

We evoked pharmacologically isolated $GABA_A$ receptor-mediated IPSCs by stimulating locally within the lateral aspect of the CeA through a bipolar stimulating electrode placed near (<100 μm) the recording electrode, while superfusing the glutamate receptor blockers 6-cyano-7-nitroquinoxaline-2,3-dione (CNQX, 10 μ*M*) and DL-2-amino-5-phosphonovalerate (APV, 30 μ*M*), and the $GABA_B$ receptor blocker CGP 55845A (CGP, 1 μ*M*). To confirm that the IPSCs were mediated by $GABA_A$ receptors, at the end of most experiments we applied the $GABA_A$ receptor antagonist bicuculline (30 μ*M*) to verify block of the IPSCs. We also used a paired-pulse facilitation (PPF) protocol with an interstimulus interval of 50 msec and stimulus strength adjusted such that the amplitude of the first IPSC of the pair was 50% of maximal amplitude determined from input/output (I/O) relationships. We took measures before ethanol (control), during ethanol (5–15 min), and after ethanol washout (20–30 min) to calculate the amplitude of IPSCs or of the ratio between the second and first IPSCs. We express all values as mean ± SEM. We subjected data to a between-subjects or within-subject ANOVA with repeated measures and the Newman-Keuls post hoc test with $p < 0.05$ considered statistically significant. When appropriate, we used Student's paired or unpaired *t*-test.

### Miniature IPSCs (mIPSCs)

For verification of presynaptic effects via mIPSC analysis, we examined two separate sets of CeA neurons using whole-cell recording either under infrared videomicroscopic guidance or in the "blind patch" configuration[38]. All recordings were made in the presence of 10 μ*M* CNQX, 30 μ*M* APV, 1 μ*M* CGP, and 1 μ*M* tetrodotoxin (TTX; to block action potential-driven spontaneous synaptic currents). All $GABA_A$ mIPSC recordings were made with electrodes filled with an internal solution containing the following (in m*M*): 135 KCl, 10 HEPES, 2 $MgCl_2$, 0.5 EGTA, 5 ATP, and 1 GTP (the latter two added fresh on the day of recording), pH 7.2–7.3. The osmolarity of the solution was 275–290 mOsm. We analyzed the mIPSC data using Mini 5.1 software (Synaptosoft, Decatur, GA). Ethanol and CRF effects on frequency and amplitude within individual CeA neurons were evaluated using cumulative probability analysis[39], with statistical significance across grouped cells determined using a paired *t*-test ($p < 0.05$ considered significant).

### Chemical and Drugs

We purchased DNQX and APV from Tocris Cookson (Ellison, MO), bicuculline from Sigma, ethanol from Remet (La Mirada, CA), and rat/human (r/h) CRF from American peptide (Sunnyvale, CA). To avoid loss of ethanol by evaporation, we diluted the ethanol in gassed ACSF from sealed stock solutions of reagent-grade 95% ethyl alcohol in water immediately before administration. CGP 55845A was a gift from Novartis Pharma (Basel). $Astressin_2$-B, $stressin_1$, and murine urocortin 3 (mUcn 3) were gifts from Jean Rivier and Wylie Vale (Salk Institute), and NIH-3, or LWH-63 (4-ethyl-[2,5,6-trimethyl-7-(2,4,6-trimethylphenyl)-7*H*-pyrrolo[2,3-*d*]pyrimidin-4-yl]amino-1-butanol), an analog of antalarmin, was synthesized by Dr. K. Rice, as previously described[40]. We found that equal concentrations (10 μ*M*) of NIH-3 in the CeA of mice and antalarmin in rat CeA (Roberto et al., in preparation) were required to block a maximal concentration of CRF and ethanol.

## RESULTS

### CRF and Ethanol Dose Dependently Augment CeA IPSCs in Wild-Type and $CRF_2$-Knockout, but not $CRF_1$-Knockout, Mice

For analysis of the effects of r/hCRF and ethanol on GABAergic neurotransmission in the CeA, we prepared brain slices from $CRF_1$ knockout (KO) ($Crhr1^{tm1Klee}/Crhr1^{tm1Klee}$; [41]) or $CRF_2$ KO ($Crhr2^{tm1Klee}/Crhr2^{tm1Klee}$; [42]) mice and their wild-type (WT) littermates on a mixed C57BL/6J × 129 background, as well as from C57BL/6J mice. Because we found that both r/hCRF and ethanol effects were equivalent in WT littermates and C57BL/6J mice, we have pooled the data for these two groups (hereafter referred to as WT mice).

To quantitate baseline effects of CRF and ethanol on evoked pharmacologically isolated $GABA_A$ergic IPSCs, and to estimate maximal and half-maximal (apparent $EC_{50}$) concentrations for subsequent tests, we performed concentration-response analyses of CeA neurons in slices taken from WT mice. Fig. 1 shows representative examples of the extent, time course, and concentration-response relationships obtained for r/hCRF and ethanol. CRF clearly increased the amplitude of the evoked IPSCs, with an apparent $EC_{50}$ of about 9 n*M* (Fig. 1C). As reported previously for rat[5] and mouse[1] CeA, ethanol increased the amplitude of $GABA_A$ IPSCs, with an apparent $EC_{50}$ of about 12 m*M* (Fig. 1D). The logistic curve fits show roughly sigmoid shapes, as would be expected of simple ligand-receptor interaction. Notably, the maximal extent of augmentation of the mean IPSCs is about the same (~40%) for both CRF and ethanol.

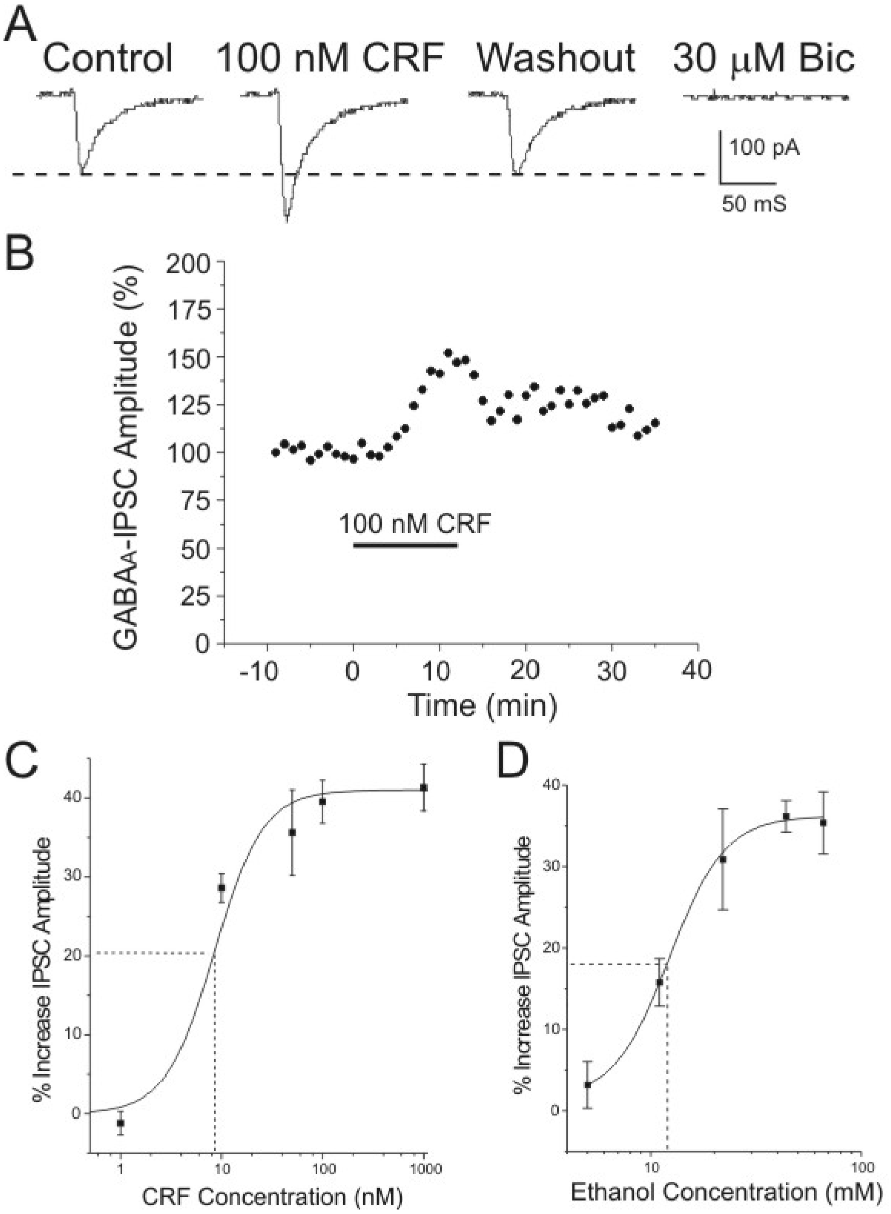

**FIGURE 1.** CRF response (A), time course of action (B), and concentration-response relationships for augmentation of IPSCs by CRF (C) and ethanol (D) superfused onto neurons in CeA slices from WT mice. (A) Representative effect of 100 n*M* CRF superfusion on IPSCs of a medial CeA neuron; note the augmentation of IPSC size by CRF, with the usual recovery on CRF washout. Subsequent bicuculline (Bic) superfusion totally blocks the IPSC, indicating mediation by $GABA_A$ receptors. (B) Time course of CRF action in another CeA neuron, showing a slow recovery from CRF after washout. (C and D) Pooled concentration-response data from five to eight neurons for each point. Points represent percent increase in mean (± SEM) IPSC peak amplitudes plotted against a log scale of drug concentrations. Solid curves are logistic fits of the data plotted by Origin software (ver. 7, OriginLab Corp, Northampton, MA), using y = (A1 – A2)/[1 + *(x/xo)p* + A2], where A1 is the initial value of IPSC increase (0–1%), A2 the estimated final maximum value (41% for CRF, 36% for ethanol), *xo* is the center *x* (8.5 for CRF; 12.3 m*M* for ethanol) value (unfixed), and *p* is the rate or power (1.8 for CRF; 3.3 for ethanol). All values were fixed except rate/power. Dashed lines indicate the apparent $EC_{50}$ for each agent: 9 n*M* for CRF and 12 m*M* for ethanol. Note the similar maximal effect of both agents. Values for the ethanol concentration-response curve taken from the dataset reported in Nie et al.[1].

In CeA neurons from WT mice, superfusion of 100 n*M* CRF, a maximally effective concentration, significantly ($F(1,9) = 6.96$, $p < 0.05$; n = 9) increased the amplitudes of $GABA_A$ergic IPSCs to 130–146% of control (Figs. 2A and B), equivalent to the CRF effects we reported previously[1]. In a separate set of eight neurons from WT mice, superfusion of 44 mm ethanol (a maximally effective concentration) also significantly ($F(1,14) = 7.32$, $p < 0.05$) enhanced the IPSCs to 131–142% of control (Figs. 2A and E). Neither CRF nor ethanol had any effect on holding currents or series resistance.

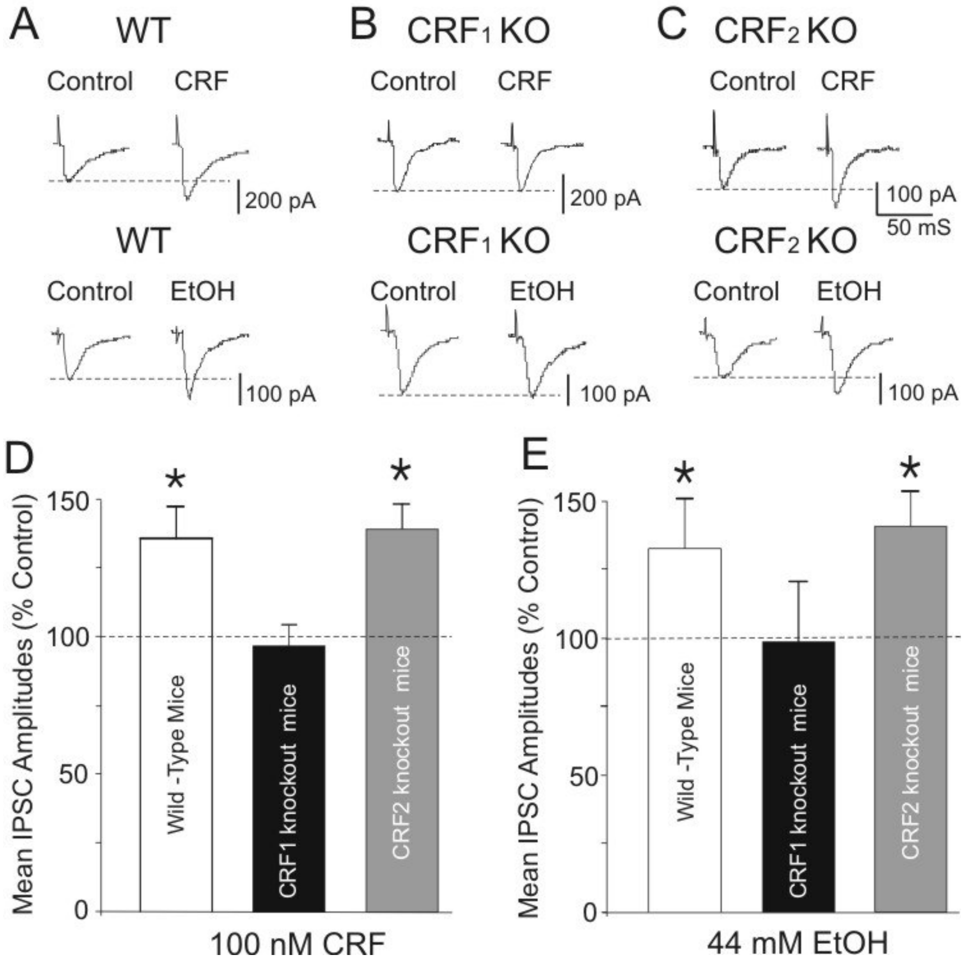

**FIGURE 2.** Both CRF and ethanol augment evoked GABAergic IPSCs in CeA neurons from WT and $CRF_2$ KO mice, but not those from $CRF_1$ KO mice. (A–C) Representative current records of individual evoked $GABA_A$ IPSCs in CeA neurons from WT, $CRF_1$ KO, and $CRF_2$ KO mice. (A) Both 100 n*M* CRF and 44 m*M* ethanol increased the amplitudes of evoked IPSCs in two different CeA neurons from WT mice. (B) Superfusion of CRF and ethanol had no effect on evoked IPSCs in CeA neurons from $CRF_1$ KO mice. (C) By contrast, superfusion of both CRF and ethanol augmented evoked IPSCs in CeA neurons from $CRF_2$ KO mice. (D) Pooled data of the effect of 100 n*M* CRF on the mean amplitudes of $GABA_A$ IPSCs. CRF 100 n*M* significantly ($p < 0.05$) enhanced the mean IPSC amplitude to 139 ± 8% of control in neurons from WT mice (n = 8) and to 139 ± 7% in neurons from $CRF_2$ KO mice (n = 6), but had no significant ($p > 0.05$) effect on mean IPSC amplitude in neurons from $CRF_1$ KO mice (n = 6). (E) Pooled data of the effect of 44 m*M* ethanol on mean $GABA_A$ IPSC amplitudes. Ethanol significantly ($p < 0.05$) augmented the mean IPSC to 137 ± 13% of control in neurons from WT mice (n = 7) and to 141 ± 9% in CeA neurons from $CRF_2$ KO mice (n = 7), but had no significant ($p > 0.05$) effect in these neurons from $CRF_1$ KO mice (n = 11). * = $p < 0.05$.

We have also seen little evidence for desensitization of the CRF or ethanol effects either in rat (Roberto et al., in preparation) or mouse CeA (see also [1]). Indeed, in some cases, the CRF effect was difficult to "wash out" after a long exposure (Fig. 1B). To further explore this issue, we performed ethanol-CRF interaction studies using intracellular recording of WT mouse CeA. When we applied a maximal concentration (44 m*M*) of ethanol as control, followed by ethanol plus maximal CRF (200 n*M*), there was a further, but insignificant ($p = 0.12$, paired *t*-test; n = 5), increase in IPSC amplitude (EtOH alone, 120 ± 12%; EtOH + CRF, 148 ± 23%; washout, 108 ± 14%). Such lack of summated effects (partial occlusion) would be expected if the two ligands act at least partially through the same mechanism. Similarly, when maximal CRF was superfused first, and then CRF plus maximal ethanol, a nonsignificantly greater mean IPSC amplitude was obtained than with CRF alone (CRF, 122 ± 3%; CRF + EtOH, 141 ± 12%; washout, 102 ± 8%; n = 7, $p < 0.14$ by paired *t*-test). We tentatively interpret the apparent, but insignificant, summation as a possible indication of activation of, or allosteric changes in, $CRF_1$ receptors by ethanol.

We also re-examined the effects of CRF and ethanol on GABAergic transmission in CeA slices taken from $CRF_1$ KO mice (see also [1]). As shown in Figs. 2B and D, superfusion of 100 n*M* CRF had no significant effect on IPSCs in seven cells from the $CRF_1$ KO mice ($p > 0.05$). As reported[1], in another 11 cells from $CRF_1$ KO mice, superfusion of 44 m*M* ethanol also had no significant ($p > 0.05$) effect on the IPSCs (Figs. 2B and E). There were no differences between the amplitudes or shapes of GABAergic IPSCs evoked in the CeA of WT mice compared to $CRF_1$ KO mice (see Figs. 2A and B). Because both CRF and ethanol enhanced GABAergic IPSCs in CeA slices from WT mice, but not $CRF_1$ KO mice, we conclude that their effects involve $CRF_1$ receptors.

To determine if the enhancement of GABAergic IPSCs might also involve $CRF_2$ receptors, we have further examined the effect of CRF and ethanol on CeA neurons from $CRF_2$ KO mice. In six cells from these KO mice, superfusion of 100 n*M* CRF significantly ($F(1,10) = 6.17$, $p < 0.05$) increased GABAergic IPSC amplitudes to 132–141% of control (Figs. 2C and D), a level equivalent to that for the CeA of WT mice. In another six CeA neurons from $CRF_2$ KO mice, superfusion of 44 m*M* ethanol also significantly ($F(1,12) = 8.57$, $p < 0.05$) increased the IPSC amplitudes to 132–144% of control (Figs. 2C and E), also equivalent to that in WT mice. There was no difference in the baseline size or shape of IPSC amplitudes in WT mice compared to $CRF_2$ KO mice. These data suggest that $CRF_2$ receptors are not necessary for the r/hCRF or ethanol enhancement of GABAergic IPSCs in mouse CeA.

## The $CRF_1$ Agonist $Stressin_1$, but not the $CRF_2$ Agonist mUcn 3, Augments Evoked IPSCs

To investigate further the role of $CRF_1$ receptors in IPSCs from mouse CeA, we applied $stressin_1$, a selective synthetic peptide agonist for $CRF_1$[29,43], by superfusion onto slices from WT C57BL/6J mice. In five CeA neurons, 1 μ*M* $stressin_1$ significantly ($F(1,12) = 8.15$, $p < 0.05$) increased GABAergic IPSC amplitudes to 135–141% of control (Figs. 3A and B). As with r/hCRF, $stressin_1$ had no effect on holding currents in CeA neurons.

To assess further whether $CRF_2$ receptors might be involved in the r/hCRF effect, we examined the effect of the natural, highly selective, $CRF_2$ agonist mUcn 3[44] on the CeA IPSCs. Superfusion of 1 μ*M* mUcn 3 had no effect on evoked IPSC amplitudes in any of the seven CeA neurons studied from WT mice (Figs. 3A and B). This negative finding also was obtained in slices in which Ucn 3 was applied first or in $stressin_1$-naïve neurons. These data support the hypothesis that r/hCRF and $stressin_1$ augment GABAergic IPSCs in CeA neurons via activation of $CRF_1$.

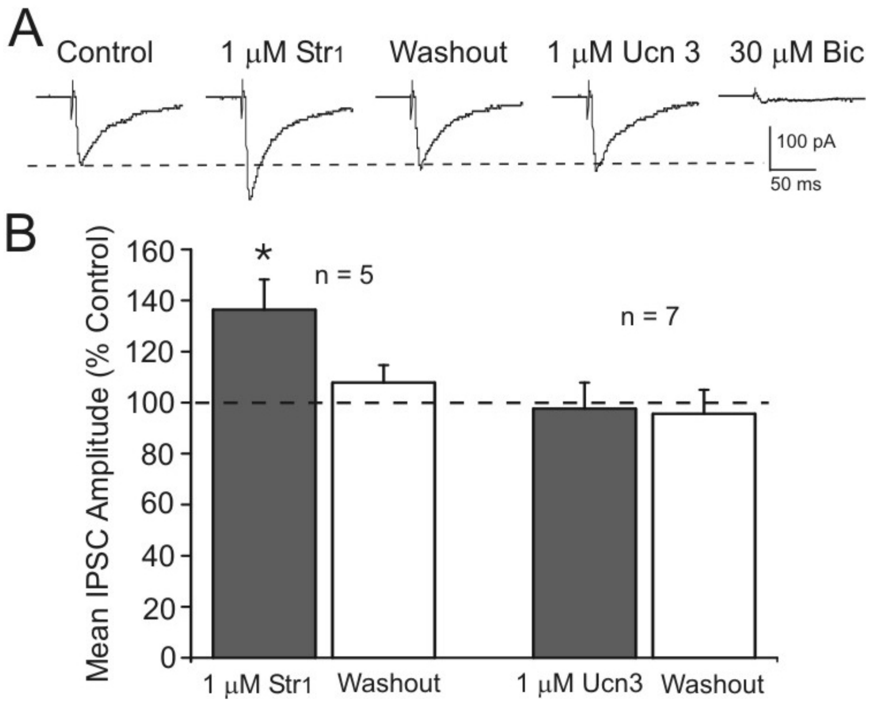

**FIGURE 3.** Stressin$_1$, but not Ucn 3, augments evoked GABAergic IPSCs in CeA neurons from WT mice. (A) Representative current traces of evoked IPSCs from a CeA neuron. Superfusion of 1 μ*M* stressin$_1$ (Str$_1$, a selective CRF$_1$ agonist) increases the amplitude of evoked GABAergic IPSCs in this CeA neuron, with recovery to control levels on washout. In the same neuron, superfusion of 1 μ*M* Ucn 3 (a selective CRF$_2$ agonist) has no effect on the IPSCs. Subsequent bicuculline (Bic; 30 μ*M*) superfusion totally blocked the IPSCs, indicating that they were mediated by GABA$_A$ receptors. (B) Pooled data: 1 μ*M* stressin$_1$ significantly ($p < 0.05$) increased the mean amplitudes of evoked GABA$_A$ergic IPSCs in five CeA neurons from WT mice, with recovery to control levels on washout. However, 1 μ*M* Ucn 3 had no significant effect on mean evoked IPSCs in seven CeA neurons from WT mice. * = $p < 0.05$.

## A Selective CRF$_1$, but not CRF$_2$, Antagonist Blocks Ethanol and CRF Effects in CeA Neurons

We also determined whether CRF$_1$ or CRF$_2$ antagonists could block the CRF or ethanol augmentation of GABAergic IPSCs. In six of six cells from WT mice, superfusion of the selective nonpeptide CRF$_1$ antagonist NIH-3 (LWH-63; 10 μ*M*) totally blocked the CRF-induced increase of evoked IPSC amplitudes in CeA slices (Figs. 4B and C). In seven of seven CeA neurons from WT mice, superfusion of 10 μ*M* NIH-3 also totally blocked the usual ethanol-elicited increase of IPSC amplitudes in CeA neurons (Figs. 4A and B; compare to Fig. 2). Superfusion of 10 μ*M* NIH-3 alone had little measurable effect on GABAergic IPSCs (Figs. 4A and B). Although 10 μ*M* might seem to be a high NIH-3 concentration (see above for similar findings with the CRF$_1$ antagonist antalarmin), unpublished studies of rat CeA (Roberto et al., in preparation) indicate that the selective CRF$_1$ antagonist R121919, that is more water-soluble than NIH-3 or antalarmin, totally blocks CRF effects at 1.0 μ*M*, as does the peptide antagonist D-phe-CRF12-41 at 200 n*M*[1]. Further, the lack of CRF$_2$ involvement in the r/hCRF and ethanol augmentation of evoked GABAergic IPSCs suggested by the KO and agonist data above is supported further by our published data[1] showing that the selective CRF$_2$ antagonist astressin$_{2\text{-}B}$[45] had no effect on either the CRF- or ethanol-induced increase of IPSC amplitudes. Thus, the combined antagonist data further support the hypothesis that ethanol augmentation of evoked GABAergic IPSCs involves CRF$_1$, but not CRF$_2$, receptors in the mouse CeA.

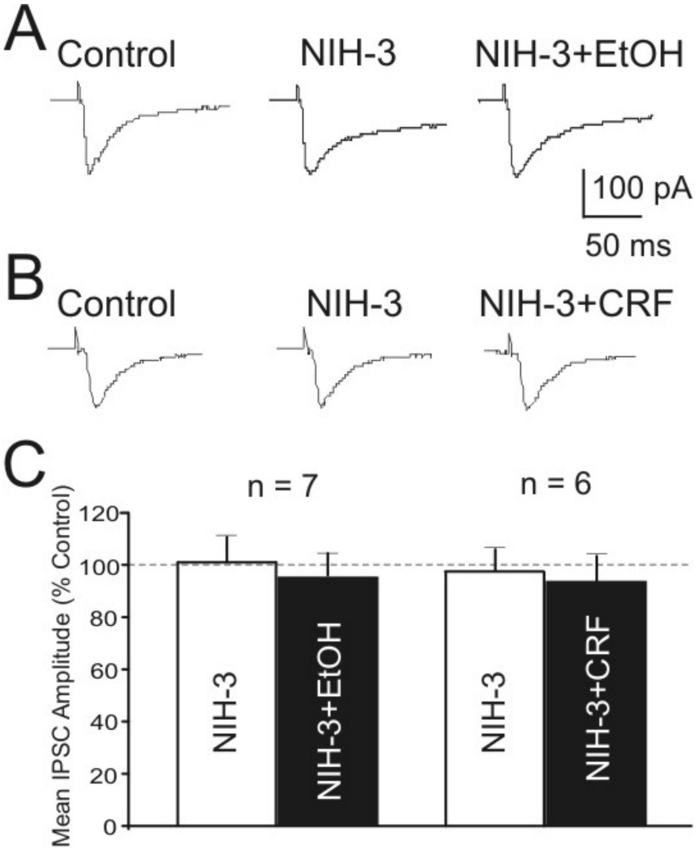

**FIGURE 4.** A selective $CRF_1$ antagonist blocks ethanol and CRF effects on evoked IPSCs in CeA neurons. (A) Representative current records of evoked IPSCs from a CeA neuron. Superfusion of the $CRF_1$ antagonist NIH-3 (10 µ*M*) alone has little direct effect on the IPSCs, but blocks the usual ethanol (A; 44 m*M*) and CRF (B; 100 n*M*) augmentation of evoked IPSCs in this CeA neuron from a WT mouse. (B) Pooled data of the effect of NIH-3 with ethanol or CRF on $GABA_A$ IPSCs in CeA neurons from WT mice (n = 5). NIH-3 (10 µ*M*) had no direct effect on the mean evoked IPSC amplitude, but significantly prevented the usual ethanol and CRF effects on mean amplitudes (compare to Figs. 1 and 2).

## CRF and Ethanol Act at a Presynaptic Site to Increase IPSCs in Mouse CeA

Because CRF and ethanol could act at either pre- or postsynaptic sites to enhance IPSC size, we extended our previous studies of the PPF of GABAergic IPSCs (using an interstimulus interval of 50 msec, previously shown to be the most sensitive to ethanol effects[5,8]). In six of six CeA neurons from WT mice, superfusion of 100 n*M* CRF or 44 m*M* ethanol significantly decreased PPF of GABAergic IPSCs (Figs. 5A and C, left panel), consistent with our previous findings[1]. This effect suggests an increased GABA release from presynaptic terminals, because changes in PPF are known to be inversely related to transmitter release (i.e., a reduction of PPF is associated with an increased probability of transmitter release). By contrast, in four CeA cells from $CRF_1$ KO mice, superfusion of 44 m*M* ethanol had no significant ($p > 0.05$) effect on the PPF of GABAergic IPSCs (Figs. 5B and C), suggesting that ethanol acts presynaptically via activation of $CRF_1$ receptors. There was no evidence for a consistent difference in baseline PPF between CeA IPSCs of $CRF_1$ KO and WT mice.

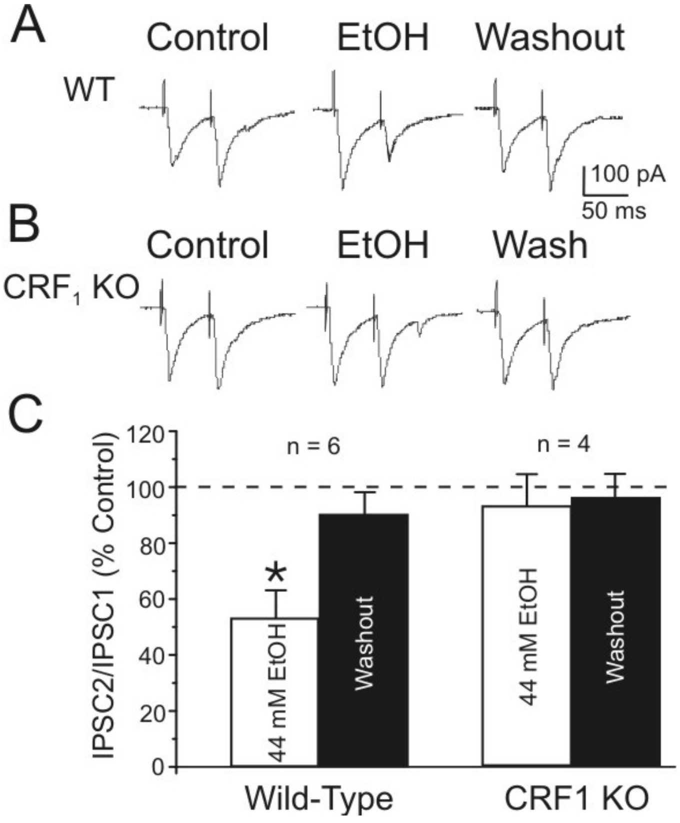

**FIGURE 5.** Ethanol decreases the PPF of evoked $GABA_A$ IPSCs (paired-pulse IPSCs evoked with interstimulus intervals of 50 msec) in CeA neurons from WT mice, but does not significantly effect PPF of IPSCs in CeA neurons from $CRF_1$ KO mice. (A) Representative current records of evoked IPSCs. Superfusion of 44 m*M* ethanol decreases the paired-pulse ratio of IPSCs (IPSC2 over IPSC1), to become paired-pulse inhibition, in a CeA neuron from a WT mouse, with recovery on washout. (B) Current records of evoked IPSCs: In a CeA neuron from a $CRF_1$ KO mouse, superfusion of 44 m*M* ethanol has little effect on the PPF of evoked IPSCs. (C) Pooled data comparing the effect of 44 m*M* ethanol on mean paired-pulse ratios of IPSCs from WT and $CRF_1$ KO mice. Ratios are expressed as percent of mean baseline value (100%). Ethanol 44 m*M* significantly decreased the mean PPF of IPSCs from WT mice, but had no significant effect on the PPF of evoked IPSCs from $CRF_1$ KO mice. * = statistical significance at $p < 0.05$ via ANOVA.

To further demonstrate that r/hCRF acts at presynaptic sites to enhance IPSC size, we recorded pharmacologically isolated, spontaneous mIPSCs in 1 μ*M* TTX. In six visually identified CeA neurons from WT mice, superfusion of 100 n*M* CRF significantly ($p < 0.05$) enhanced the frequency of mIPSCs (Figs. 6A and B) in the CeA from WT mice, but had little effect on the amplitude of mIPSCs (Figs. 6A and C). To confirm that this r/hCRF effect involves $CRF_1$, we tested the selective $CRF_1$ antagonist NIH-3. Superfusion of NIH-3 (10 μ*M*) alone only slightly decreased the frequency of mIPSCs in CeA slices. However, NIH-3 significantly inhibited the r/hCRF enhancement of mIPSC frequency (data not shown), suggesting that presynaptic $CRF_1$ receptors mediate the CRF enhancement of GABAergic synaptic transmission in mouse CeA neurons.

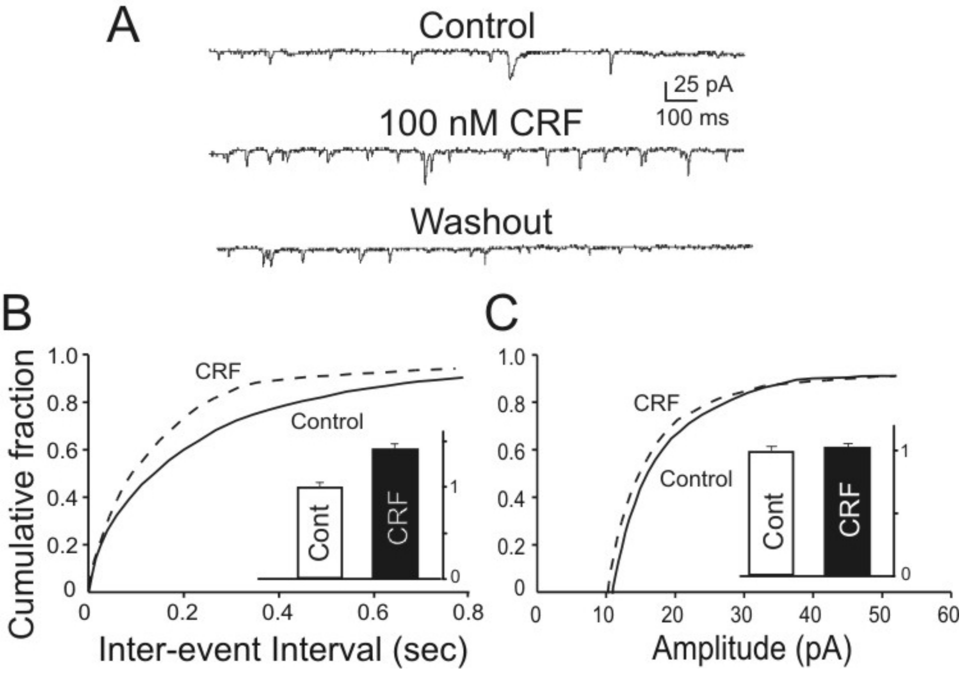

**FIGURE 6.** CRF enhances the frequency of spontaneous $GABA_A$ergic mIPSCs in CeA neurons from WT mice. (A) Representative current traces recorded from a CeA neuron in the presence of 1 μ*M* TTX and glutamate blockers. Superfusion of 100 n*M* r/hCRF clearly increases the frequency of the mIPSCs, with little obvious effect on their amplitude, with recovery on washout. Number of recorded events: Control, 9; CRF, 23; washout, 13. (B) Cumulative frequency histograms for a representative CeA neuron from WT mice showing a shift to the left, indicating briefer inter-event intervals (higher frequencies) during the application of 100 n*M* r/hCRF. (C) Cumulative amplitude histograms from the same CeA neuron showing little r/hCRF effect on the distribution of mIPSC amplitudes in CeA neurons from WT mice. Inset graphs are pooled data from six CeA neurons, showing that mean mIPSC frequency is significantly ($p < 0.05$) increased, but mean amplitude is not changed by CRF.

To minimize possible sampling bias of the visually identified neurons, we tested another set of CeA neurons using the "blind slice" or "blind patch" configuration. In these five CeA neurons, superfusion of 200 n*M* CRF significantly ($p < 0.05$) increased the frequency, but not the amplitude, of the mIPSCs (Figs. 7A–C), with recovery to control frequencies on washout of CRF (Fig. 7A). The findings suggest a lack of visual or cell-morphology bias in the sampling of CeA neurons with regard to the apparent presynaptic effects of CRF in increasing GABA release in CeA.

In another set of five CeA neurons, superfusion of 44 m*M* ethanol also significantly ($p < 0.05$) enhanced the frequency of mIPSCs in all five CeA neurons from WT mice (Figs. 8A and B), but only slightly and insignificantly ($p > 0.05$) decreased the amplitude of mIPSCs (Figs. 8A and C) in two cells, suggesting that ethanol increases GABAergic synaptic transmission in mouse CeA predominantly by a presynaptic mechanism, as in the rat CeA[5]. Superfusion of the $CRF_1$ receptor antagonist NIH-3 (10 μ*M*) blocked the ethanol-induced increase of the frequency of mIPSCs in three cells (Fig. 8D), further indicating a role for presynaptic $CRF_1$ receptors in the ethanol augmentation of IPSCs.

## DISCUSSION

These combined data suggest that an endogenous $CRF_1$ agonist and presynaptic $CRF_1$ receptors are involved in the ethanol augmentation of GABAergic transmission in CeA neurons. Thus, in the present studies, we

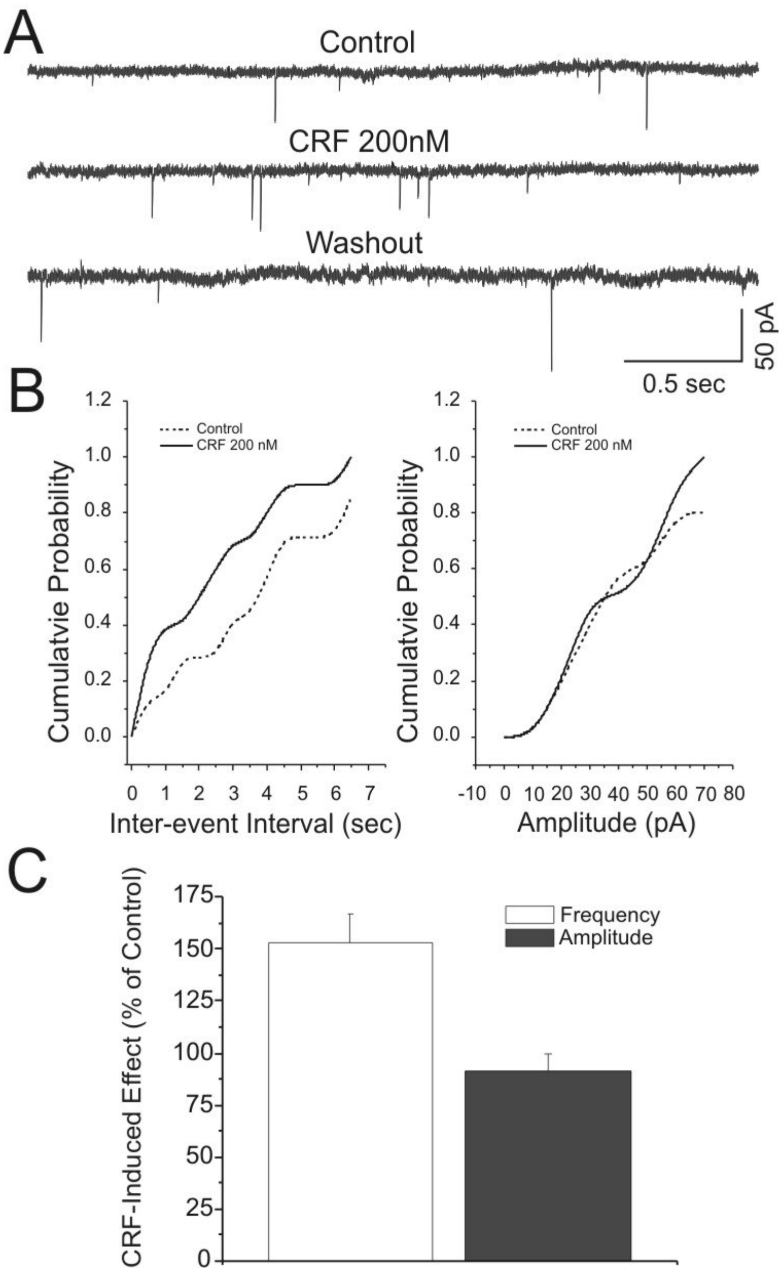

**FIGURE 7.** CRF increases the frequency of mIPSCs in WT murine CeA neurons recorded using the "blind patch" whole-cell configuration. All recordings performed in the presence of glutamate (DNQX 20 μ*M* and APV 30 μ*M*) and $GABA_B$ (CGP 1 μ*M*) receptor antagonists and of 1 μ*M* TTX. (A) Representative current traces of mIPSCs from a CeA neuron with low baseline mIPSC frequency, showing that superfusion of 200 n*M* CRF (3 min) increased mIPSC frequency, with recovery after 9 min of washout (bottom trace). This neuron was held at –60 mV throughout the experiment. Number of recorded events: Control, 9; CRF, 14; washout, 6. (B) Cumulative event histograms of the same CeA neuron as in (A). Left panel: CRF increased the cumulative probability of mIPSCs with short inter-event intervals (greater frequency), with no effect on mIPSC amplitudes (right panel). (C) Pooled data from five WT mouse CeA neurons; 200 n*M* CRF significantly ($p = 0.002$) increased mean mIPSC frequency (by over 50%), but had no significant effect on mean mIPSC amplitude.

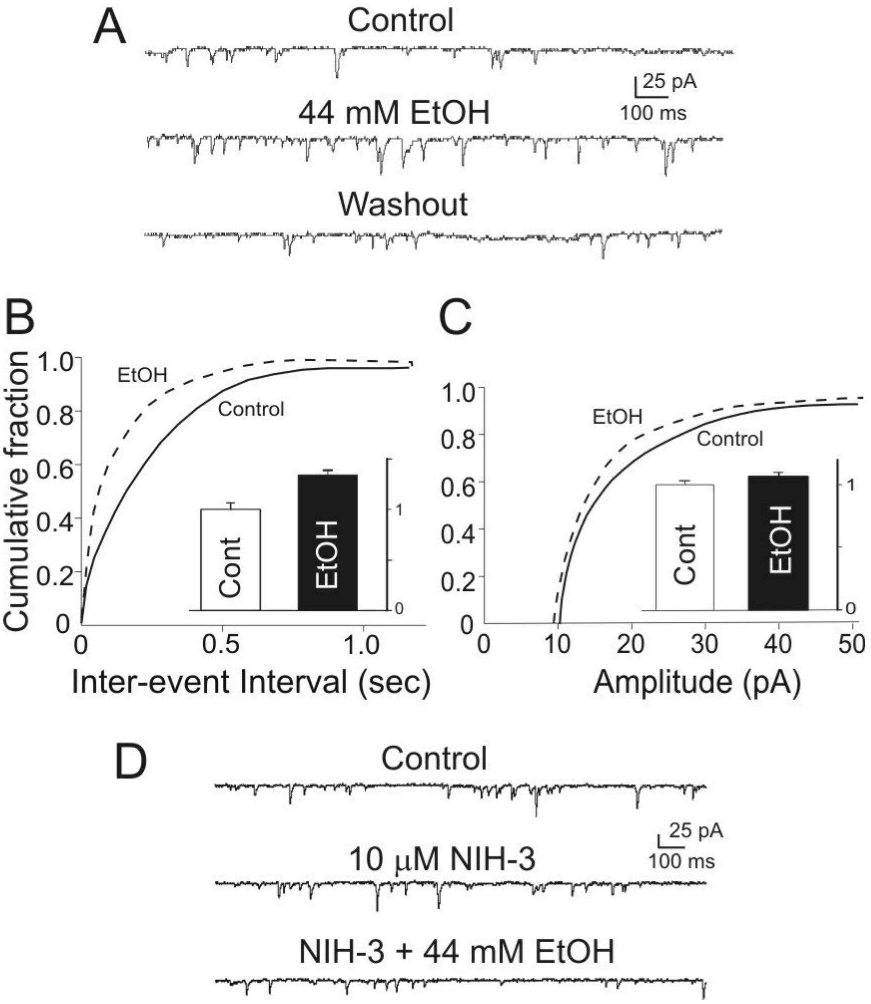

**FIGURE 8.** Ethanol increases the frequency of spontaneous mIPSCs in CeA neurons from WT mice. (A) Representative current traces recorded from a CeA neuron in the presence of glutamate blockers and TTX. Superfusion of 44 m*M* ethanol increases the frequency, but has little effect on the amplitude, of mIPSCs, with recovery on washout. Number of recorded events: Control, 14; ethanol, 31; washout, 17. (B) Cumulative frequency histogram of mIPSCs from a representative CeA neuron showing a shift to the left with ethanol, indicating a short inter-event interval (higher frequencies) during the application of 44 m*M* ethanol. (C) Cumulative amplitude histogram from the same CeA neuron showing a small, but insignificant ($p$ = 0.32), decrease in the distribution of mIPSC amplitudes. Inset graphs in B and C are pooled data from six CeA neurons showing that mean mIPSC frequency is significantly increased ($p$ = 0.027), but mean amplitude is only increased insignificantly ($p$ = 0.56) by ethanol. (D) Current traces from another CeA neuron of a WT mouse. Superfusion of the $CRF_1$ antagonist NIH-3 (10 μ*M*) alone has little effect on mIPSCs, but blocks the ethanol enhancement of IPSC frequency in this neuron. Number of recorded events: Control, 22; NIH-3, 18; washout, 15.

have verified that sedating and intoxicating concentrations of ethanol augment GABAergic synaptic transmission in the CeA from WT and $CRF_2$ KO mice, but have virtually no effect on CeA GABAergic synaptic transmission in $CRF_1$ KO mice[1]. Further, in the present study, the selective $CRF_1$ agonist stressin$_1$, but not the $CRF_2$ agonist Ucn 3, augmented GABAergic transmission; also the selective $CRF_1$ antagonist NIH-3 blocked the ethanol enhancement of GABAergic synaptic transmission in WT mice, but the $CRF_2$ antagonist astressin$_{2\text{-}B}$ had no effect on this enhancement. Both CRF and ethanol decreased PPF and increased the frequency of mIPSCs in CeA neurons from WT mice, but not the PPF in the CeA of $CRF_1$ KO mice, suggesting a role for presynaptic $CRF_1$ receptors in the ethanol effect. Because these effects of ethanol on evoked IPSCs and PPF were completely absent in the CeA of $CRF_1$ KO mice, and ethanol effects on evoked and mIPSCs were blocked by the $CRF_1$ antagonist in the CeA of WT mice, presynaptic $CRF_1$ receptor activation appears to be necessary for these ethanol effects. Although little is known yet about the exact molecular mechanism(s) underlying the $CRF_1$ mediation of ethanol action, the effect appears to be action-potential independent. It is possible that ethanol increases GABA release from presynaptic terminals by triggering release of endogenous CRF that, in turn, activates $CRF_1$ receptors on GABAergic terminals to enhance quantal vesicular GABA release. Alternatively, ethanol could act directly on presynaptic $CRF_1$ receptors to increase GABA release[2].

GABA is the major inhibitory neurotransmitter in the CNS, and enhancement of GABAergic activity is a common property of many sedative and hypnotic drugs, including ethanol[2,3,4,5,46]. Although many previous behavioral and neurochemical studies focused on postsynaptic $GABA_A$ receptors as important targets for ethanol action in the CNS, more recent electrophysiological studies have suggested that ethanol can also act on GABAergic terminals to increase GABA release presynaptically[2,5,9]. Behavioral studies have shown that injection of a $GABA_A$ agonist into the CeA decreases ethanol self-administration in dependent rats[36]. Our lab recently reported electrophysiological data showing that ethanol increased GABAergic transmission at both pre- and postsynaptic sites in rat CeA neurons[5], and that ethanol significantly augmented evoked GABAergic IPSCs in mouse CeA neurons via a $CRF_1$ mechanism[1]. The present findings extend the mouse data to verify a presynaptic vesicular site of ethanol action in the mouse and further implicate activation of presynaptic $CRF_1$, but exclude requirement for $CRF_2$ receptors, in this ethanol effect.

The CRF system plays an important role in the regulation of anxiety-related behavior, and is implicated in anxiety and depressive disorders[13]. The biological actions of endogenous CRF and its structurally related paralogs (Ucns 1, 2, and 3) are mediated by two subtypes of G-protein-coupled receptors, $CRF_1$ and $CRF_2$, each with different splice variants, expression patterns, and physiological functions[14,47,48]. r/hCRF shows high $CRF_1$ and only moderate $CRF_2$ affinity, Ucn 1 acts on both $CRF_1$ and $CRF_2$ with high affinity, and Ucns 2 and 3 bind $CRF_2$ receptors selectively. Several lines of behavioral evidence point to the participation of $CRF_1$ receptors in mediating the stress-related effects of CRF. For example, $CRF_1$-deficient mice show reduced anxiety-related behavior[41,49], and administration of $CRF_1$, but not $CRF_2$, antisense oligodeoxynucleotides[50] or antagonists[50] also reduce anxiety-related behaviors. These and other data suggest that changes in the activity of $CRF_1$ receptor systems are involved in stress-related psychiatric disorders.

Much emerging evidence indicates an interaction between stress, brain CRF and GABA systems, and alcohol drinking[51,52,53,54]. Notably, the CRF system in the amygdala appears to be activated during stress and ethanol withdrawal, as evidenced by the increased CRF release measured by *in vivo* microdialysis[19], with resulting tissue content depletion[33]. The GABA system has provided a model for many anxiolytic pharmaceuticals, such as the benzodiazepines[55]. More recently, several studies have focused on the interaction between CRF and GABA. Interestingly, in the paraventricular nucleus (PVN), GABA appears to inhibit CRF secretion tonically[56]. Conversely, our studies suggest that $CRF_1$ receptor activation increases GABA release in the CeA. Although there is a reduction in extracellular GABA in the amygdala with conditioned fear stressors[57], exposure to predator stress increases CRF release in the PVN, and both CRF and GABA release in the amygdala[58]. In the latter study, injection of a CRF or $GABA_A$ receptor antagonist into the amygdala immediately prior to stress had only a small effect on early stress responses, but significantly altered responses to repeat stress administered 2 days later, suggesting that release of both CRF and GABA in the amgydala is involved in plasticity following stress responses.

## CONCLUSIONS

Our data indicate that CRF regulates or enhances GABA release from presynaptic terminals in the CeA slice. As GABA and CRF are colocalized in about half of the (mostly GABAergic) neurons in the

CeA[59], $CRF_1$ receptors could play a role as feedback autoreceptors that enhance GABA release from presynaptic terminals. However, the reported[60] low levels of mRNA for $CRF_1$ within the CeA may suggest that the increased GABA release could arise from activation of $CRF_1$ receptors on GABAergic terminals that arise from extrinsic sources, such as the basolateral amygdala or cortical amygdaloid nuclei. By increasing GABAergic inhibition of the CeA GABAergic interneurons, ethanol may disinhibit downstream components of the extended amygdala, such as the bed nucleus of stria terminalis.

As noted above, both CRF and $GABA_A$ receptors are likely involved in anxiety and depression[61,62], and both disorders are implicated in excessive alcohol drinking in humans. Dysregulation of $CRF_1$ receptors may contribute to several stress-induced psychiatric disorders, such as human alcoholism[53,63], and numerous reports suggest that stressful life events and maladaptive responses elicit alcohol drinking and relapse behavior[64]. However, the molecular and cellular mechanisms underlying stress-induced alcohol drinking are still unknown. Our findings that both CRF and ethanol presynaptically enhance GABAergic transmission in a brain region known to be involved in stress-related behaviors provide a possible mechanism that links stress and depression/anxiety with ethanol reinforcement. Thus, our data support the hypotheses that CRF plays a crucial role in some behaviors associated with ethanol and that presynaptic $CRF_1$ receptors represent an important therapeutic target for the treatment of stress-related alcohol drinking.

## ACKNOWLEDGMENTS

We thank Drs. G. Koob, P. Schweitzer, and S. Moore for helpful comments; T. Acosta for technical support; Drs. J. Rivier and W. Vale (Salk Institute) for the gift of $stressin_1$ and Ucn 3; Drs. K.-F. Lee and W. Vale (Salk Institute) for the receptor null mice; and Drs. W. Fröstl and A. Suter (Novartis Pharma) for the gift of CGP-55845A. This work was supported by NIH grants U01-AA10994 (under the INIA Consortium of the NIAAA), AA06420, DA03665, and NS38633. This work also was partially supported by the NIH Intramural Research Programs of the National Institute on Drug Abuse, the National Institute of Alcohol Abuse and Alcoholism, and the National Institute of Diabetes and Digestive and Kidney Diseases.
